# Supplementary material for: Complex‐centric proteome profiling by SEC‐SWATH‐MS
Source: Mol Syst Biol. 2019 Jan 14;15(1):e8438. doi: 10.15252/msb.20188438 (PMC6346213; doi:10.15252/msb.20188438)
Supplement: Supplementary file 8 — Dataset EV7 [file MSB-15-e8438-s008.zip › feature_plots_string/Q15942.pdf]

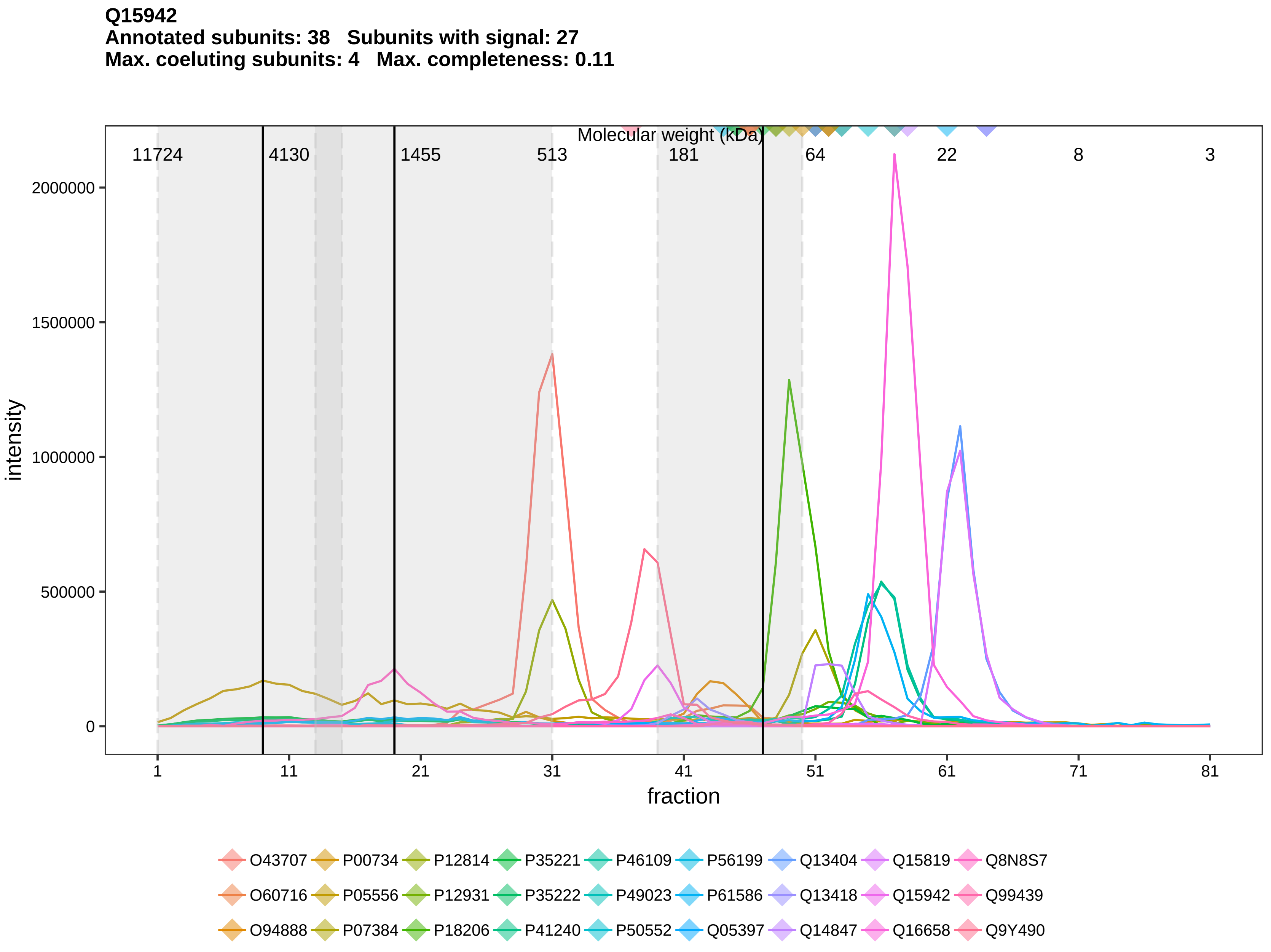
Q15942
Annotated subunits: 38   Subunits with signal: 27
Max. coeluting subunits: 4   Max. completeness: 0.11
Molecular weight (kDa)
11724
4130
1455
513
181
64
22
8
3
intensity
2000000
1500000
1000000
500000
0
fraction
1
11
21
31
41
51
61
71
81
O43707
P00734
P12814
P35221
P46109
P56199
Q13404
Q15819
Q8N8S7
O60716
P05556
P12931
P35222
P49023
P61586
Q13418
Q15942
Q99439
O94888
P07384
P18206
P41240
P50552
Q05397
Q14847
Q16658
Q9Y490
